# Editorial: Natural Compounds and Novel Sources of Antimicrobial Agents for Food Preservation and Biofilm Control

**DOI:** 10.3389/fmicb.2022.856858

**Published:** 2022-03-30

**Authors:** Lizziane Kretli Winkelströter, Eugenia Bezirtzoglou, Fabricio Luiz Tulini

**Affiliations:** ^1^University of Western São Paulo, Presidente Prudente, Brazil; ^2^Democritus University of Thrace Komotini, Komotini, Greece; ^3^Federal University of Western Bahia Barreiras, Barreiras, Brazil

**Keywords:** biofilms, foodborne pathogens, food safety, biocontrol, food additives

Biofilms can be defined as highly structured sessile microbial communities, embedded in an extracellular polymeric substance, which allows irreversible adherence to biotic and abiotic surfaces. In nature, biofilms can be formed by numerous species of bacteria, fungi, protozoa, and algae. Among the species of microorganisms commonly involved in the formation of biofilms are *Staphylococcus aureus, Staphylococcus epidermidis, Pseudomonas aeruginosa, Escherichia coli*, and *Candida albicans* (Joshi et al., 2021; Srinivasan et al., 2021). The ability to form biofilms determines the pathogenicity of these microorganisms. Currently, it is believed that 80% of human bacterial infections are associated with biofilm formation, especially those involving the use of medical devices, such as catheters, heart valves, contact lenses among others (Algburi et al., 2017; Joshi et al., 2021; Srinivasan et al., 2021).

The development of microbial biofilms also occurs frequently in the food industries, since there is a large amount of nutrients available in equipment, utensils and contact surfaces. Biofilms have a higher resistance to sanitizers and can lead the equipment to corrosion, causing a negative impact on the quality of the final product. Its presence in food and processing can cause serious damage to public health due to problems associated with foodborne diseases and food deterioration (Mevo et al., 2021).

The production of safe and high-quality food has become a challenge for the food industry due to food deterioration, which is caused by the undesirable growth of pathogenic/deteriorating microorganisms. This deterioration can lead to food loss and waste. To reduce losses, the food industry uses synthetic preservatives to control microbial growth in order to extend shelf life, quality, and food safety. However, some chemical additives when ingested in large quantities can cause undesirable reactions to consumers. Nowadays, it has been observed an increased consumer preference for more natural food additives and concern about the safety of synthetic preservatives that have encouraged the food industry to look for environmentally friendly alternatives. Thus, the intention arose to replace the use of traditional synthetic preservatives with natural antimicrobials in food (Mei et al., 2019; Yu et al., 2021).

In addition, the indiscriminate use of antimicrobials, including antibiotics and biocides, has led to the development of resistance in food and hospital bacteria. These resistances pose a threat to public health, as they reduce the efficacy of the same compounds, thus increasing mortality and morbidity (Srinivasan et al., 2021). Microorganisms in the form of biofilms become substantially more resistant to the action of antibiotics than those living alone. This fact can be attributed to the weak penetration and diffusion of antimicrobial drugs through the extracellular polymer matrix, strong expression of efflux pumps and enzymes capable of degrading antimicrobial molecules (Algburi et al., 2017). These mechanisms have driven several studies in an attempt to develop materials resistant to the adhesion of microorganisms, in addition to possible treatments in the case of biofilms already formed.

Thus, it is essential to study new antimicrobial compounds in the control of growth and dissemination of pathogens. Compounds of natural origin have been presented as promising alternatives to antimicrobial agents currently used, not only as possible food preservatives, but also as adjuvants in the process of disinfection of surfaces and in the fight against infections.

This Research Topic aims to address several natural agents with antimicrobial action and/or antibiofilm focused on food preservation and biofilm control, i.e., phytochemicals, biosurfactants, antimicrobial peptides and microbial enzymes, together with their sources, mechanism of action by interference in quorum detection pathways, and also, interruption of the extracellular polymeric substance, and its inhibiting concentrations. We had successfully received 19 submitted manuscript that followed the revision process and finally, it was concluded in 13 approved articles.

Review article create a readable synthesis of the best resources available in the literature for an important current area of research. In the present Research Topic, four reviews were published and contributed to suggest new research directions, to strengthen support for existing theories and/or identify patterns among existing research studies. Oulahal and Degraeve focused on a review that provided an overview of current knowledge regarding the promises and the limits of phenolic-rich plant extracts for food preservation and biofilm control on food-contacting surfaces. Khorshidian et al. investigated the possible application of pediocin (Pediocins, which belong to subclass IIa of bacteriocin characterized as small unmodified peptides with a low molecular weight, are produced by some of the *Pediococcus* genus bacteria), in preservation of meat and meat products against *L. monocytogenes*. Maurya et al. developed a review that aimed to present the practical application of nanoemulsions (a) by addressing their direct and indirect (EO nanoemulsion coating leading to active packaging) consistent support in a real food system, (b) biochemical actions related to antimicrobial mechanisms, (c) effectiveness of nanoemulsion as bio-nanosensor with large scale practical applicability, (d) critical evaluation of toxicity, safety, and regulatory issues, and (e) market demand of nanoemulsion in pharmaceuticals and nutraceuticals along with the current challenges and future opportunities. Das et al. developed a review that deals with the advancement in nanoencapsulation-based edible coating of essential oil with efficient utilization as a novel safe green preservative and develops a green insight into sustainable protection of fruits against fungal- and mycotoxin-mediated quality deterioration.

Several studies (nine original research) have addressed this theme and proved the potential application of natural and alternative components in food preservation and biofilm control. Many plants have been known to exert antimicrobial properties due to their content of secondary metabolites. Over the past decade, much attention has been placed on the study of phytochemicals for their antimicrobial activity (Barbieri et al., 2017). In the present Research Topic Santos et al. developed an alcohol-free high-performance extractive approach to recover antibacterial and antioxidants phytochemicals from red propolis. The authors found inhibition halo on the growth of *Staphylococcus aureus* and *Salmonella* enteritidis bacteria. Khelissa et al. prepared ruthenium (II) cationic water-soluble complex by a reaction between dichloro (para-cymene) ruthenium (II) dimers and aminooxime ligands in a 1:2 molar ratio. Antibacterial and antibiofilm activities of the synthetized complex were assessed against *Escherichia coli, Staphylococcus aureus, Listeria monocytogenes*, and *Enterococcus faecalis*. The results revealed that the ruthenium (II) complex has higher antibacterial and antibiofilm activities in comparison with free ligands or the enantiopure (R)-limonene.

Phenolic compounds are natural substances that can be obtained from plants and play an important antimicrobial effect. In the present Research Topic, Santos et al. aimed to evaluate the potential of phenolic compounds for QS inhibition in a QS biosensor strain (*Chromobacterium violaceum*) and three foodborne bacterial species (*Aeromonas hydrophila, Salmonella enterica* serovar Montevideo, and *Serratia marcescens*). Those authors found that curcumin, capsaicin, and resveratrol inhibited violacein production by *C. violaceum*. Biofilm formation was inhibited by resveratrol in *A. hydrophila*, by capsaicin and curcumin in *S*. Montevideo and by resveratrol and capsaicin in *S. marcescens*.

Microbial secondary metabolites are low molecular mass products, not essential for growth of the producing cultures, but very important for human health. They include antibiotics, antitumor agents, cholesterol-lowering drugs, and others (Allemailem, 2021). In the Research Topic, Kumari et al. aimed to assess potential of biosurfactants screened from a novel yeast and their inhibition against food spoilage fungi. Authors illustrated the antifungal activity of sophorolipid biosurfactant from Metschnikowia genus for the first time and suggested a novel antifungal compound against food spoilage and human fungal pathogens. Chen et al. hypothesized that deinococcal cellular constituents play a pivotal role in preventing *S. aureus* colonization by inhibiting biofilm formation. Theirs experiments proved that DeinoPol is a key molecule in the negative regulation of *S. aureus* biofilm formation by *D. radiodurans*. Therefore, DeinoPol could be applied to prevent and/or treat infections or inflammatory diseases associated with *S. aureus* biofilms.

In this scenario, Taggar et al. demonstrated that an antimicrobial peptides (named as peptide-Ba49) isolated from *Bacillus subtilis* subsp. *spizizenii* strain exhibited strong antibacterial efficacy against *S. aureus* ATCC 25923. The first article of this topic, Zhang et al. evaluated the efficacy of mixtures of natural antimicrobial compounds, namely reuterin, microcin J25, and lactic acid, for reducing the viability of *Salmonella enterica* and total aerobes on broiler chicken carcasses. They found that sprayed onto chilled chicken carcasses, this reuterin + lactic acid mixture reduced *Salmonella* spp. counts. The synergy of reuterin with lactic acid or microcin J25 as inhibitors of bacterial growth was significant. Choyam et al. demonstrated that *Bacillus* antimicrobial peptide (BAMP) produced by *Bacillus paralicheniformis* exhibited a bacteriostatic effect on *Salmonella typhi* and controls the viability of *Listeria monocytogenes* in chicken meat efficiently.

Nanotechnology is increasingly used to target bacteria as an alternative for food safety and biofilm control (Vinci and Rapa, 2019). In this Research Topic Puranen et al. evaluated the efficacy of nanomaterials and blue light illumination for *L. monocytogenes* ATCC 7644 biofilm inactivation. The results found by the authors demonstrated that nanocoating with visible light illumination could be an effective and safe method for enhancing food safety in food processing facilities to control biofilm formation.

In summary, this Research Topic provides a better understanding of the main natural and alternative components that exhibit action in the food preservation and/or in the biofilm control of pathogenic species. The results obtained from the use of these compounds have aroused great interest. There is an expectation that further *in vitro* and *in vivo* studies will be conducted to better understand their metabolic pathways, mechanisms of action, to define details about its safety and to develop regulations for its use. In this way, we hope that this Research Topic can generate knowledge and open ways for the construction of new systems and strategies to combat biofilms and food contamination.

## Author Contributions

LW, EB, and FT edited the topic and wrote the manuscript. All authors listed have made a substantial contribution to the work and approved it for publication.

## Conflict of Interest

The authors declare that the research was conducted in the absence of any commercial or financial relationships that could be construed as a potential conflict of interest.

## Publisher's Note

All claims expressed in this article are solely those of the authors and do not necessarily represent those of their affiliated organizations, or those of the publisher, the editors and the reviewers. Any product that may be evaluated in this article, or claim that may be made by its manufacturer, is not guaranteed or endorsed by the publisher.
